# Agile CD22 nanoclusters run rings around fenced BCR

**DOI:** 10.15252/embj.201593745

**Published:** 2016-01-08

**Authors:** David Depoil, Michael L Dustin

**Affiliations:** ^1^ Kennedy Institute of RheumatologyThe University of OxfordOxfordUK

**Keywords:** Immunology, Signal Transduction

## Abstract

B lymphocytes are key players in host defence, but also autoimmune diseases. Their survival depends upon tonic signals transduced by surface immunoglobulin (BCR) and the process leading to antibody secretion is initiated by interaction of BCR with a cognate antigen. CD22 limits signalling of the BCR to strike a balance between tonic signalling, reactivity to pathogens and prevention of autoimmunity. In this issue, Gasparrini *et al* (2016) combined super‐resolution imaging approaches with single‐particle tracking and simulations to show how CD22 controls the signalling state of the BCR. They demonstrated that small CD22 nanoclusters run rings around the BCR in confined steady state to maintain low tonic signals, but releasing BCR from these corrals allows BCR cluster growth, which overcomes the harrying inhibition from highly mobile CD22.

Membrane organisation plays an important role in the physiology of cells. The fluid mosaic model of membranes emphasised the importance of diffusion, which allows membrane components to interact (Singer & Nicolson, 1972). One mechanism to compartmentalise the membrane is to use actin “fences” to cordon off small fields; membrane proteins can diffuse rapidly within a field and occasionally can hop across the fence into an adjacent field (Kusumi *et al*, 2012). Batista and colleagues have previously demonstrated that the BCR signalling is held to a basal tonic signalling level by being contained by actin fences and that breaking down these fences causes robust signalling. These observations were made with single‐molecule tracking of BCR in relation of F‐actin bundles such that the confinement of BCR to the small actin‐fenced fields was directly observed. Super‐resolution microscopy based on reconstructing images from many single‐molecule observations further demonstrated that BCRs and CD19, an activating receptor that functions as an adaptor protein, are found in separate nanoclusters within the fields (Mattila *et al*, 2013; Maity *et al*, 2015). These nanosclusters are confined by cortical F‐actin bundles, but are not themselves F‐actin dependent. In fact, actin depolymerisation allows BCR nanoclusters to join together into larger clusters with CD19, leading to signalling. A tetraspanin network holds the CD19 nanoclusters together. The perturbation of this tetraspanin network will inhibit BCR triggering. Both membrane organisation and diffusion of receptor/co‐receptor maintains tonic signaling and allows BCR triggering only when the BCR encounter its cognate ligand (Treanor *et al*, 2010; Mattila *et al*, 2013).

An inhibitory pathway including the src family tyrosine kinase Lyn and its substrate CD22 tightly controls BCR signalling. Once phosphorylated by Lyn, CD22 recruits SHP‐1, a tyrosine phosphatase capable of inhibiting the tyrosine kinase cascade downstream of the BCR. CD22 has a α‐2,6 sialic actin‐specific lectin domain that is important for its inhibitory function, but the reason for this was not proven. As an aside, CD22 also appears to be able to interact in trans across the immunological synapse (Macauley & Paulson, 2014), but the impact of this type of CD22 recruitment is not explored here. Gasparrini *et al* (2016) shine a new light on how the nanoscale organisation and diffusion of CD22 parallels that of the BCR, but the same type of changes that enhance BCR signalling, larger clusters that diffuse slower, result in loss of inhibitory function for CD22.

They first show by using super‐resolution imagining techniques that, like IgM, IgD and CD19, CD22 is predominantly organised in preformed nanoscale clusters in resting B cells. These nanoclusters are in closer association with BCR than CD19. Nonetheless, by doing colocalisation studies, they found that a majority of BCR are not in association with CD22 at any snapshot in time. So to understand how CD22 could influence BCR, they developed an *in silico* model to predict the area CD22 nanoclusters could survey as a function of their diffusion. The models predicted that if CD22 diffuses at 0.05 μm^2^/s, it would cover 90% of the cell surface in 500 ms, this would explain how CD22 inhibits the signalling of multiple BCR effectively by diffusing fast enough that even with the relatively small numbers the phosphatase activity can be recruited at any location in a field (Fig 1A). When they analysed by single‐particle tracking the behaviour of CD22, they found that CD22 diffuses at a rate of 0.046 μm^2^/s that would be in agreement with the model. They demonstrate as well that the diffusion rate of CD22 confers its inhibitory role by using a mutant form (CD22 R130E) that cannot bind α2,6‐sialic acid. This mutant forms small clusters that diffuse faster than the wild‐type form (Fig 1B). As a result, the authors show that this mutant processes a stronger inhibitory function and inhibits calcium flux in B cell after actin depolymerisation. Thus, the smaller clusters formed by CD22 R130E make it even more agile.

**Figure 1:**
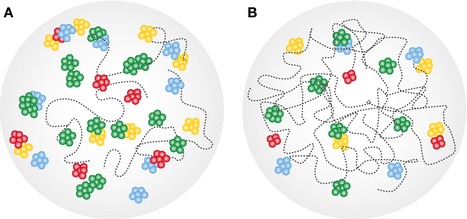
Smaller CD22 nanoclusters cover field better Fast diffusion of CD22 nano clusters allows CD22 inhibitory function even if outnumbered by BCR and CD19 nanoclusters. (A) IgM (yellow), IgD (green), CD19 (blue) and CD22 (red) are organised as nanocluster at the surface of B cells. CD22 clusters are outnumbered by “activatory” signalling clusters. Their high‐speed diffusion allows them to “visit” and sample multiple activating clusters thus allowing CD22 inhibitory function. (B) The mutation of the sialic acid binding capacity of CD22 reduces the size of the CD22 cluster and increases their diffusion speed as well as the area cover. This results in a stronger inhibitory signal.

These results provide a first look at how the large class of negative regulatory receptors that recruit SHP‐1 or SHP‐2 may function. This includes the NK inhibitory receptors, which in NK cells also undergo rapid diffusion and nanoclustering (Pageon *et al*, 2013), and PD‐1, which recruits SHP‐2 to act as a checkpoint inhibitor of T cells (Yokosuka *et al*, 2012). It will be interesting whether the concept described by Gasparrini *et al* (2016) will also apply to this entire class of inhibitory receptors that the control of signalling by rapid diffusion in small fields bounded by F‐actin fences, but that this regulation can be overcome by breakdown of F‐actin barriers or when ligands generate larger clusters of activating receptors.
